# The semantics, sociolinguistics, and origins of double modals in American English: New insights from social media

**DOI:** 10.1371/journal.pone.0295799

**Published:** 2024-01-24

**Authors:** Cameron Morin, Jack Grieve

**Affiliations:** 1 Department of English, Ecole Normale Supérieure de Lyon, Lyon, France; 2 Department of English Language and Linguistics, University of Birmingham, Birmingham, United Kingdom; The University of Lahore, PAKISTAN

## Abstract

In this paper, we analyze double modal use in American English based on a multi-billion-word corpus of geolocated posts from the social media platform Twitter. We identify and map 76 distinct double modals totaling 5,349 examples, many more types and tokens of double modals than have ever been observed. These descriptive results show that double modal structure and use in American English is far more complex than has generally been assumed. We then consider the relevance of these results to three current theoretical debates. First, we demonstrate that although there are various semantic tendencies in the types of modals that most often combine, there are no absolute constraints on double modal formation in American English. Most surprisingly, our results suggest that double modals are used productively across the US. Second, we argue that there is considerable dialect variation in double modal use in the southern US, with double modals generally being most strongly associated with African American Language, especially in the Deep South. This result challenges previous sociolinguistic research, which has often highlighted double modal use in White Southern English, especially in Appalachia. Third, we consider how these results can help us better understand the origins of double modals in America English: although it has generally been assumed that double modals were introduced by Scots-Irish settlers, we believe our results are more consistent with the hypothesis that double modals are an innovation of African American Language.

## 1 Introduction

Double modals (DMs), which are sequences of two modal verbs (e.g. *I* ***might can*** *go to work*), have long attracted the attention of linguists studying the English language. DMs have been observed in some dialects of American English, primarily in the South, and more rarely in Scots, including Borders Scots [1] and Ulster Scots [2], as well as several English-based creoles including Gullah, Jamaican Creole, and Guyanese Creole [3]. Although they are commonly labelled ‘double modals’, there is a long-standing debate on the exact grammatical nature of these features, which have often been analysed as adverb-modal sequences or single modal lexical items [4]. Theoretical research on DMs has focused on their syntactic status [5–7], semantic constraints on their formation [8–11], their regional and social distribution [12, 13], and their historical origins [14–16].

In addition to their unusual structure and marked social meaning, DMs are of interest because they are one of the most uncommon grammatical constructions recognized in the English language. The infrequency of DMs, however, has greatly limited their empirical analysis. Standard multi-million-word corpora are simply too small to reliably observe such rare constructions, even in dialects where DMs are most commonly found. The study of DMs has therefore largely been based on data collected through forms of elicitation, including acceptability judgments [17], dialect surveys [18], and sociolinguistic interviews [19]. But such approaches to data collection are limited by a lack of natural observation. For example, because DMs are so rarely used or even heard by most linguists, it is difficult to know which DMs to focus on, what range of DMs is possible, and how best to elicit these forms, when collecting data from informants.

Our limited record of DM use has led to two major gaps in our understanding of DMs. First, we do not know the full inventory of DMs or which combinations of modals are most common. Given the rarity and potential complexity of these forms, this type of description can only be obtained through the analysis of very large corpora of natural language. Most notably, this gap makes it difficult to assess semantic constraints on DM formation. Second, we know relatively little about dialect variation in DM use. Sociolinguists and dialectologists have collected data from a relatively small number of informants, mainly in the American South [20–22], but this data has not been of sufficient quantity to allow for detailed mapping or forms of quantitative sociolinguistic analysis. This gap makes it difficult to understand what factors condition the regional and social distribution of DMs or explain their historical origins.

Fortunately, the massive increase of language online has begun to allow for DMs to be studied at scale for the first time [13], opening new lines for descriptive and theoretical research that were unimaginable 50 years ago [23]. Our goal in this study is therefore to conduct the largest ever description of DM use in American English based on a very large corpus of Twitter data, and to consider how these results inform current theoretical debates about DMs in linguistics. Specifically, this paper pursues five main research questions, including two descriptive research questions and three theoretical research questions.

First, we answer two descriptive research questions through a quantitative corpus analysis of a 8.9 billion word corpus of geolocated Twitter posts collected from across the contiguous US between 2013 and 2014:

**(1)** What is the inventory and frequency distribution of DMs on American social media?
**(2)** What is the regional distribution of DMs, individually and in the aggregate, as used on American social media?

Second, based on our empirical results, we consider three basic and open theoretical research questions related to the semantics, sociolinguistics, and history of DMs:

**(3)** What are the semantic factors that constrain DM formation in American English?
**(4)** What are the social and regional factors that condition variation in the use of DMs in American English?
**(5)** What are the historical origins of DMs in American English?

Although our study is based solely on modern social media data, we believe that our unique perspective on DM use, especially the large number of DM types and tokens we have observed and mapped across the counties of the contiguous US for the first time, allows us to make important contributions to our general theoretical understanding of DMs, challenging long held assumptions about DMs in American English.

The rest of the paper is organized as follows. In Section 2, we present a review of previous research on DMs, especially in American English, focusing on research in semantics and syntax, dialectology and sociolinguistics, and historical linguistics. In Section 3, we introduce our corpus and discuss the strengths and weaknesses of this approach to data collection. In Section 4, we present our main descriptive analysis, providing answers to our two descriptive research questions. In Section 5, we consider our empirical results in light of our three theoretical research questions. Finally, in Section 6 we conclude by summarizing our main findings and considering the relevance of our results for future research on DMs.

## 2 Background

### 2.1 Double modal meaning and structure

DMs are rare grammatical constructions in the English language involving a sequence of two modal verbs in a single tensed clause [12]. More generally, they are considered a type of *multiple modal*, which can consist of a sequence of more than two modals [14]. The following examples from DiPaolo [24] and the Yale Grammatical Diversity Project [12] are seen as typical DMs in American English:

(1) *I don’t think I have any grants you* ***might could*** *apply for*.
*We* ***might can*** *go up there next Saturday*.
*I* ***may could*** *at Finger’s*.
*You know, if you drank half a drink, you* ***might oughta*** *go home and sleep it off*.
*This thing here I* ***might should*** *turn over to Ann*.
*How is it no one* ***might not would*** *notice that but Anne?*
*Well, once we get under way, it* ***shouldn’t oughta*** *take us very long*.

DMs are considered non-standard constructions and assumed to be absent from most dialects of English. Despite being unfamiliar to many English speakers, the meaning of DMs can generally be inferred. For example, in first position, *might* and *may* have a similar meaning as the adverb *maybe*, whereas, in second position, *could* and *ought to* have a similar meaning as the semi-modals *be able to* and *have to*.

The few studies that have analyzed the semantics of DMs in detail generally agree DM combinations are constrained by an Epistemic-to-Root ordering with the first modal having an *epistemic meaning*, reflecting the speaker’s degree of confidence in the proposition, and with the second modal having a *root meaning* (deontic or dynamic), reflecting the speaker’s attitude regarding the possibility of the subject being involved in the event [8, 25, 26]. More rarely, both modals can fall on the same end of this spectrum. As for the broader pragmatic meaning of DMs, Mishoe and Montgomery [9] and DiPaolo [19] argue that DMs are used for one-on-one negotiation, face saving, and hedging, while Hasty [10, 11] describes how DMs can be used by doctors for mitigation in their interaction with patients. There is also general agreement that DMs with *may* and especially *might* in first position are most acceptable and that these two modals rarely occur in second position [7, 24, 27]. More specifically, Hasty [20] claims there is a general DM acceptability hierarchy, with *might oughta* being most acceptable, followed by *might could, might should* and *might would*, where a speaker who finds one form acceptable will find all DMs higher in the hierarchy acceptable as well.

There has been more research on the grammar of DMs, especially to account for their challenging syntactic structure. The existence of DMs has even been questioned, with much debate around whether these constructions involve two true modals [4]. For example, in a 2007 *Language Log* post, “Do double modals really exist?”, Pullum questioned the status of DMs:

*I think there might be no double modals at all*. *I think it might be just a matter of the emergence of a small number of new adverbs with a rather strong preference for being used before certain modals*.

In addition to their rarity, the syntactic status of DMs has been questioned because they are difficult to explain within formal frameworks, if only because of the one tensed-verb per clause constraint generally thought to hold for English [28]. For example, Nagle [4] argues that DMs are problematic for standard generative theories, where modals are treated as syntactic heads of verb phrases. There have been several proposals for the analysis of DMs within the generative framework: DMs have been viewed as two sister verb constituents [29], single lexical items [24], adverb–modal sequences [5], and two-clause predicates [4]. More recently, studies have analyzed DMs as interactions between embedded modals with slightly different features [6, 7], with Hasty developing a minimalist treatment of DMs in American English [7, 10, 11, 17, 20, 30]. These studies have presented increasingly complex accounts of DMs to account for the modal-like behavior of both elements and the one-tensed-verb constraint—a challenge that has not yet been definitively resolved. Similar challenges have been acknowledged in other frameworks, such as Generalized Phrase Structure Grammar and Lexical-Functional Grammar [4, 31, 32].

### 2.2 Double modal inventory and variation in American English

In this section, we review available inventories of DMs proposed in the literature, highlighting empirical limitations of previous research, and the lack of an observational baseline. Linguists have observed a range of DMs across dialects of the English language [15, 16]. DMs are found in some dialects of English spoken on the British Isles, including Borders Scots [33–35] and Tyneside English [36]; they may also be acceptable in Northeastern Northern Ireland where Ulster Scots is spoken [2]. The most frequently attested DMs in the British Isles as reported in the literature are *might can*, *will can*, *used to could*, and *must can* [37, 38]. Other DMs that are less frequently discussed are *might could* [33], *should can* and *have to can* [37], and *would could* [36, 39].

DMs, however, are most frequently attested in dialects of American English, especially in the South. The two traditional dialect surveys that covered this region, the *Linguistic Atlas of the Middle and South Atlantic States* (LAMSAS) [40] and the *Linguistic Atlas of the Gulf States* (LAGS) [18], observed DMs across the South, from Texas to Pennsylvania [12]. Numerous studies have also examined DMs in parts of the South, including Alabama [21], the Carolinas [9, 23, 25], Tennessee [7, 17, 20, 41], Texas [19, 24, 29, 42, 43], and West Virginia and Appalachia [22, 44]. In addition, DiPaolo [24] observed DM use in Utah, and, more recently, Collins and Singler [45] observed the use of the otherwise unattested DM *would might* by a young white man without a southern accent in New York City.

There has therefore been a rather wide variety of regions in which DMs have been attested. The majority of DM forms presented in previous research have been collected in the *Multimo Database* [46]. Overall, this database contains 2,030 tokens of American DMs spanning 30 types involving 9 central modals (*can, could, may, might, shall, should, will, would, must*) and 2 semi-modals (*ought to, used to*). The most common types are *might could*, *might can*, *might would*, *used to could*, and *may can* (see Table 1). It should be noted that despite the value of this dataset, the relative frequencies of DM must be interpreted with care, as the vast majority of the tokens were collected through elicitation across multiple studies spanning several decades and states using various approaches to data collection.

**Table 1:** DMs in Multimo by number of positive declarative tokens.

**DM**	*Might could*	*Might can*	*Might would*	*Used to could*	*May can*	*Might ought to*	*Might should*	*May could*
**Tokens**	889	174	148	101	78	69	50	30
	*Used to would*	*Could might*	*Might will*	*May should*	*May will*	*May would*	*Would might*	*May ought to*
	23	21	19	18	18	16	9	4
	*Must can*	*Should ought to*	*Will can*	*Can might*	*May might*	*Might used to*	*Could used to*	*May shall*
	4	4	4	3	2	2	2	1
	*May used to*	*Might may*	*Must could*	*Ought to could*	*Ought to will*	*Ought to would*	*Should might*	
	1	1	1	1	1	1	1	

It is especially notable that such a small number of the possible two-way combinations of modals have been attested in American English. For example, taking the 9 primary modals and the 2 semi-modals, excluding reduplication, and ignoring variation in pronunciation/spelling (e.g. *oughta*), there are 110 potential English DMs; however, studies have never reported more than 30, with only half of these DMs being observed at least 5 times. There is also disagreement on which types are most common or even possible, aside from general agreement that *might could* is most common. For example, DiPaolo [24] lists 25 “actually occurring double modals”, including DMs involving quasi-modals (*supposed to, need to, better*), as well as the multiple modal *might woulda had oughta*. Alternatively, Hasty [20] lists the 15 most common DMs in the literature, excluding those involving quasi-modals and notably including three DMs not listed by Di Paolo (*must can, could oughta, would oughta*), while excluding three DMs listed by Di Paolo that would appear to meet Hasty’s definition (*can might, could might, oughta could*), highlighting the uncertainty surrounding this basic descriptive question.

It is unclear why the inventory of DMs varies so substantially across studies. This variation may simply reflect limitations in sample size and considerable differences in how data was collected and interpreted, but this variation may also represent regional or social dialect variation. This topic has received relatively little attention, although some preliminary observations can be made. Hasty claims that his acceptability hierarchy varies across the South [20], and adds that the full set has been observed in South Carolina [25], while subsets have been observed in Alabama [21] and West Virginia [22]. Alternatively, the maps in LAGS [9] suggest DMs occur across the Gulf States with little regional variation. Similarly, the LAMSAS data shows that DMs are concentrated in the South Atlantic States, although tokens are also observed in Pennsylvania and Maryland. Overall, our understanding of the regional distribution of DMs is therefore limited, aside from general agreement that DMs are a feature of southern dialects.

The distribution of DMs in African American Language (AAL) has also received relatively little attention. Fennell and Butters [15] claim that DMs are generally used in the South by both White and African American speakers, but elsewhere their use is restricted primarily to African Americans. Mishoe and Montgomery [9] offer data for four DMs (*might can, might could, might would, used to could*) based on LAGS data annotated for sex, social class and ethnicity, showing that DMs are generally more acceptable to African American speakers than White speakers, except for *might could*, which is also by far the most acceptable DM overall (see Table 2). *Might would* is also notable as being far more acceptable to African Americans. Table 2 also clearly shows that DMs are more acceptable to speakers from lower class backgrounds regardless of ethnicity.

**Table 2:** Incidence of four DMs in LAGS by ethnicity, sex, and class, adapted from [9].

DM	*Might can*	*Might could*	*Might would*	*Used to could*
**White overall (n/%)**	**38 (4.3%)**	**173 (19.8%)**	**20 (2.3%)**	**51 (5.8%)**
**Black overall**	**15 (6.1%)**	**46 (18.7%)**	**21 (8.5%)**	**19 (7.7%)**
White men	24 (5%)	93 (19.2%)	12 (2.5%)	29 (6%)
Black men	10 (8.9%)	24 (21.4%)	8 (7.1%)	9 (8.0%)
White women	14 (3.6%)	80 (20.5%)	8 (2%)	22 (5.6%)
Black women	5 (3.7%)	22 (16.4%)	13 (9.7%)	10 (7.5%)
White lower class	13 (6.2%)	55 (26.1%)	10 (4.7%)	21 (10%)
Black lower class	10 (7%)	33 (23.2%)	11 (7.7%)	13 (9.2%)
White middle class	24 (4.4%)	105 (19.2%)	10 (1.8%)	29 (5.3%)
Black middle class	5 (5.4%)	11 (11.9%)	9 (9.7%)	5 (5.5%)
White upper class	1 (0.9%)	13 (11.1%)	0 (0%)	1 (0.9%)
Black upper class	0 (0%)	2 (22.2%)	1 (11.1%)	1 (11.1%)

Finally, in a recent study, Coats [13] applied a corpus-based approach to study DMs in North America based on a 1.2 billion word corpus of geolocated YouTube video transcripts generated via Automatic Speech Recognition, primarily from town hall meetings but also including interviews, vlogs, and sports commentaries. After hand checking a small percentage of the possible DMs he extracted, the majority of which were false positives, Coats identified 1,035 real DM tokens, representing 67 DM types, far more than have previously been observed. Coats also mapped the frequencies of a selection of these DMs across the US at the state level, showing that DMs are concentrated in the Southeast as expected, but are somewhat more widespread. In addition, Coats mapped the precise location of all 1,035 DM tokens together, showing that although DMs are concentrated in the Southeast, there are surprisingly few tokens both in Appalachia and areas of the Deep South with high African-American populations (i.e. central Georgia, Alabama, Mississippi), where we would expect to find these DMs based on previous research. It is unclear, however, to what extent these maps are limited by variation in population density or uneven sampling across locations, registers, and social groups. Nevertheless, because he finds substantial numbers of DMs across North America, Coats concludes that DMs are “not solely restricted to specific regional dialects, but are rather an (infrequently used) resource available to most speakers of North American English for the careful expression of modality” [13, p. 3]. More recently, Coats used a similar corpus of YouTube video transcripts to study DMs in British English [47].

### 2.3 Origins of double modals

A final important line of research concerns the origins of DMs in American English, which is a highly speculative area given both the lack of historical data and our limited of understanding of contemporary DM use. The most widely accepted theory is that DMs were brought to America by the Scots-Irish, who primarily settled Appalachia in the eighteenth and nineteenth centuries, having previously moved from Scotland to Northern Ireland in the seventeenth century [48]. From Appalachia, DMs are then assumed to have spread across the South, including to people from other backgrounds [49]. The primary evidence cited to support this theory is that DM usage in the US is concentrated in areas with large Scots-Irish populations, especially Appalachia [22]. In addition, linguists have noted that *can* and *could* appear to be the most common modals in second position in both varieties [14, 50].

Although this is the main explanation provided in the literature for the origins of DMs in American English, empirical evidence is limited given how few DMs have been observed. Most notably, the fact that Appalachia has been the site of considerable research on DMs cannot be taken as direct evidence that DMs are especially common in this region. It is also difficult to compare the inventories or relative frequencies of DMs in these two dialects given the limited amount of empirical data. In fact, based on what we do know, despite some similarities, the inventories of DMs in dialects of Scots and American English appear to be different. Although *might can* and *used to could* appear to be common in both, the DMs *will can, should can, have to can*, and *would could* appear to be characteristic of Scots, while *might could, might would, may can, might ought to*, and *might should* appear to be characteristic of American English. In addition, Montgomery and Nagle [49] argue that the American DM system is set apart from the Scots DM system by a further degree of auxiliarization of the modal *can* and a greater range of combinations. The assumption is therefore that DM constructions were introduced to America by Scots-Irish settlers, but that this system changed substantially over time, for unknown reasons, as it diffused across the wider population.

It is also notable that DMs are not especially common in modern Ulster Scots, as spoken in Northern Ireland today, which is where Scots-Irish settlers originated, but rather in Borders Scots as spoken in Scotland [2, 37], although this may simply reflect change over time. This theory also does not explain how or why DMs first emerged in Scots. DMs have been hypothesized either to be an internal development of Scots or a borrowing from Scandinavian, which allowed multiple modals like other North Germanic Languages [51]. Although this is by no means counter-evidence to the theory that the Scots-Irish introduced DMs to American English, it does represent a further gap in our knowledge of the origins of DMs, pointing to a general lack of understanding about how DMs can enter dialects of the English language.

A secondary theory of DM origins in American English is that they came to the US via the Caribbean, given the presence of DMs in Caribbean creoles and Gullah [52, 53], assumed once again to have been introduced by colonists from a Scottish background, and perhaps restructured via creolization [15]. Fennell and Butters [15], however, conclude that DMs in these varieties are too different to be the main source of American DMs, and that American DMs most likely originate from Scots-Irish settlers in the US. Similarly, in a recent study, Zullo and colleagues [16] proposed the existence of a “pan-Atlantic multiple modal belt” based on MultiMo and the Electronic World Atlas of Varieties of English [3]. They identified three multiple modal clusters: British and American, Caribbean, and South Atlantic islands. Based on similarities across varieties, they suggest that multiple modals originated in Scots and were then imported into American English, where they were restructured through contact in Caribbean varieties.

Overall, the origins DMs are therefore unclear, despite general agreement that the only plausible theory is importation by Scots-Irish immigrants to the Upper South, with the primary evidence cited being the contemporary inventory and regional distribution of DMs in American English. The alternative view—i.e., that DMs are an internal innovation in American English—has been proposed, but always dismissed. For instance, Fennell and Butters [15] consider whether DMs may be true internal innovations in American English, but ultimately reject this hypothesis, primarily due to the regional distribution of DMs in American English [15].

For the sake of completeness, we need to consider a final logically possible origin for double modal constructions, namely, that they were not imported from either Britain or the Caribbean, but instead developed spontaneously in the American South. While on the surface this might seem a different explanation from the others, in effect it reduces to the Scots-origin theory, since, as we have already mentioned, the southern areas in which double modals are used were primarily settled by Scotch-Irish and northern British English dialect speakers.

[15, p. 276]

However, given that our knowledge of both the inventory and distribution of DMs in American English is problematic, greatly limited by the lack of observations of DMs in natural language, we believe such conclusions are premature and warrant further empirical investigation.

## 3 Data

### 3.1 Corpus

Despite the large amount of research on DMs, it is clear there are major gaps in our understanding of the inventory, regional distribution, semantics, sociolinguistics, and origins of DMs in American English primarily because they have been so hard to observe in natural language. Corpus data can therefore help us better understand such an elusive and potentially complex phenomenon, especially if we want to assess the frequencies of different DMs so as to establish probabilistic constraints on their structure or to study variation in their use. Traditional corpora, however, are simply far too small to allow for such rare forms to be observed at scale, and rarely represent the types of non-standard dialects and informal register that are presumably associated with the production of these forms. Furthermore, because DMs are so uncommon and socially restricted in their use, they are not very familiar to most linguists, making it difficult for linguists who study DMs through elicitation to ensure that the structure and use of these forms are studied in their full complexity.

Over the past decade, however, the rise of internet communication, especially through highly informal forms of social media, has opened up new possibilities for the analysis of DMs at scale in natural language. As can be seen through Twitter searches, there are relatively large numbers of DMs accessible online, both in terms of types and tokens. Consequently, very large corpora sampled from Twitter, or other online platforms like YouTube [13], can allow for the large-scale quantitative analysis of DMs for the first time. We therefore take advantage of this emerging data source to systematically describe DM usage on American Twitter at an unprecedented scale.

For this study, we have analyzed an 8.9-billion-word corpus of American Twitter consisting of approximately 980 million geolocated tweets from 7 million users collected between October 11, 2013 and November 22, 2014 from across the contiguous US at the University of South Carolina using the Twitter API (http://dev.twitter.com) [54]. The collection and analysis method thus complied with the terms and conditions for this platform. Although the period represented by the corpus spans 409 days, in total the corpus includes 399 days because 10 days are missing due to various technical issues that interrupted data collection. The corpus contains on average 22 million words per day, but ranges from 10 to 29 million words per day. We do not consider change over time, and whether the results of this study would replicate on this platform today is an empirical question outside the scope of this study, although we suspect that change over time in these forms over less than a decade, even online, would be relatively limited. Because each tweet is geolocated with the longitude and latitude of the user when they posted on a smartphone, this corpus is especially useful for mapping regional linguistic variation. Notably, after 2015, Twitter changed from a system where users needed to opt out of automated geolocation, leading to a precipitous drop in the amount of geolocated data available on Twitter.

The main reason we chose to use this corpus is therefore because it is the largest sample of geolocated social media data that is accessible to us—and one of the largest geolocated corpora for any variety of language that has ever been compiled for academic research—providing a unique opportunity to observe DMs at scale. Twitter is also highly informal, which appears to encourage the use of DMs compared to many other varieties, including varieties of spoken English. For example, the spoken component of the Corpus of Contemporary American English [55] contains only 162 DM tokens, despite containing over 127 million words, illustrating how rare this construction is. Our Twitter corpus has also been validated in a range of previous studies, which have mapped lexical and grammatical dialect variation in American English [54], compared the use of hesitation markers across Germanic languages [56], tracked the emergence lexical innovations in American English [57, 58], and introduced quantitative methods for modeling language change [59].

To prepare the corpus for spatial analysis, all individual posts were sorted by county (or county equivalent) based on their longitude and latitude, effectively stratifying our corpus into regionalized sub-corpora, thereby allowing for detailed spatial analysis and mapping. In total, the corpus contains 3,075 counties out of the 3,108 total counties in the contiguous US at the time (99%), with missing data primarily occurring in small, sparsely populated counties in the Central States. On average, the corpus contains 2 million words per county, but ranges from 300 to 300 million words per county. Overall, 98% of the 3,075 counties are represented by at least 10,000 words and 79% of the counties are represented by at least 100,000 words.

### 3.2 Data collection and cleaning

To identify DMs in the corpus, we automatically extracted all tweets containing sequences of any 2 of the 9 primary modals of English (*can, could, may, might, shall, should, will, would, must*) and the 2 semi-modals (*used to, ought to*). To focus on the primary phenomenon of DMs, we ignored duplicate combinations (e.g. *could could*), as well as other semi-modals (e.g. *going to/gonna*), modals followed by *have* (e.g. *woulda*), and several other related forms such as combinations involving *might as well*. We also did not consider DMs split by other words like adverbs, *not*, or pronouns involved in subject-auxiliary inversion, although this would be an interesting area for further research, especially to better understand the syntax of DMs. In total, following this procedure, we automatically extracted 10,137 potential DMs.

After these potential DMs were examined, however, it was clear that many instances were not true DMs. We therefore manually analyzed all 10,137 possible DM tokens to identify valid sequences of two modals in a single tensed clause based on an analysis of the linguistic structure of the tweets.

Obvious types of “false” DMs, which were excluded from our dataset, included forms showcased below. In (2), we see tweets where the regular expression extracted words that are not modal verbs: *may* is in fact the month of May, lacking a capital letter due to informal language use; *will* is a lexical verb and not an auxiliary (meaning ‘to wish, to desire’); *might* is a noun (meaning ‘strength’). In (3), we see clear cases of auto-correction and typing errors (3), where the modification or lack of only a couple of letters for a different word results in a form that looks like a modal: *should* is presumably meant to be *show* in the first example, *shoulder* in the second, and *would* is presumably meant to be *world* in the third. Finally, in (4), we see adjacent single modals across clause boundaries, which were not marked typographically in the tweet due to informal language use or syntactic ambiguity: in many of these cases, the authors simply did not use punctuation to signal these boundaries as is standard (e.g. *I should. Might be up all night again*; *I might, can you put me down*).

(2) *Hoping having my Spanish final on Cinco de* ***may will*** *bring me some luck*
  *I wish I* ***could will*** *myself to sleep*
  *For not by* ***might shall*** *a man prevail*
(3) *I always wonder who all* ***would should*** *up to my funeral*
  *My* ***should might*** *start peeling soon*
  *Think slowly and your* ***would will*** *blossom*
(4) *Need a nap but don’t think I* ***should might*** *be up all night again*
  *Whenever you* ***can would*** *be great!*
  *I* ***might can*** *you put me down just in case*.

After we removed all problematic tokens following this methodology, we were left with 5,439 DM tokens (54% of all possible DM tokens), which make up the main dataset for this study. Crucially, all these tokens clearly consist of two modal verbs in a row in a single tensed clause, where neither modal appears to be a typographical error. We make this full dataset available for further analysis.

### 3.3 Advantages and limitations of the corpus

Finally, before presenting our descriptive results, it is important to discuss the advantages and disadvantages of this type of social-media, big-data, corpus-based approach to linguistics, which has become especially popular in sociolinguistics, where it is known as *computational sociolinguistics* [60, 61].

There are several advantages to working with large social media corpora, especially Twitter. The main benefit, as we have highlighted, is that language can be accessed in large volumes, allowing for rare constructions like DMs to be observed at a scale that has not previously been possible. In addition, unlike many written registers, Twitter is highly informal and used by a diverse range of people, estimated to be used by around 21% of the US population at the time of data collection [62], thereby increasing the likelihood that rare, colloquial, and circumscribed constructions like DMs can be observed directly, even compared to spoken corpora, as noted above. Access to spatial metadata also allows for variation in DM usage to be mapped at high resolution. Finally, natural language corpora generally overcome the Observer’s Paradox [63], as the linguist has no involvement in the act of data production. This is especially important when analyzing highly localized forms like DMs, as they may be avoided in elicitation, especially when fieldworkers come from outside the community.

There are, however, also disadvantages to working with social media corpora. Most notably, like any corpus-based study, results can only be assumed to generalize directly to the variety that is represented by the corpus. Analyzing Twitter can only provide us with a direct picture of language use on Twitter, which is different in many ways from the spoken vernacular that is the ostensible focus of most research in linguistics. Furthermore, although Twitter is very popular, the user base differs from the general population, with slightly higher engagement from younger people, African Americans, and urban residents [62]. Social media can provide access to communities that fieldworkers may have previously under-sampled, but this approach limits access to other communities, i.e. people who are less likely to engage with social media, especially perhaps older people as well as people who live in rural areas, although we do have very good coverage across most of the US. Ultimately, however, our descriptive results are only intended to directly describe DM use on Twitter, naturally and rightfully reflecting the underlying demographics of this population of English speakers. Finally, corpus-based approaches generally make it difficult to directly interrogate fine-grained differences in the meaning and acceptability of different constructions. Elicitation is likely still necessary in these cases, although access to baseline empirical data on the inventory and frequency of DMs is generally necessary to direct this type of research.

We keep these limitations in mind as we interpret our results, including being careful to limit our descriptions to the variety under analysis. Nevertheless, at this time, Twitter is the only variety that can be sampled at the necessary scale and with the necessary metadata to allow for the type of analysis reported in this study. Furthermore, previous research on comparable corpora for British English has shown that dialect maps based on Twitter data broadly align with dialect surveys [64]. We therefore believe there is much to learn about DMs based on this corpus, not only on Twitter, but in American English more generally, especially given the lack of sufficient amounts of data from other varieties. The degree to which these results generalize is an open question, although we can begin to compare our results to previous data-driven research [13], which was based on a relatively large corpus of spoken language collected online. As more natural and informal language data becomes available, we hope the study of DMs can be further extended and generalized, including by drawing on the methods and results of this study.

## 4 Descriptive results

### 4.1 Inventory and relative frequencies of double modals

Based on the 5,439 observed DM tokens, we identified 76 distinct DM types, representing 69% of the 110 possible DM types for which we searched, with 56 DMs occurring at least 5 times. Table 3 lists all DM types found in decreasing order. To give a sense of what the tokens look like in the corpus, sample tweets are shown below containing four of the most common DMs, *might can* (5), *might could*, (6), *should would* (7), and *would could* (8) as well as two less common DMs, *will should* (9) and *would might* (10).

**Table 3:** Frequencies of DM types.

**DM**	*Might can*	*Might could*	*May can*	*Might would*	*Should would*	*Would could*	*Must can*	*Might should*
**Raw freq**.	1733	932	273	243	177	171	164	146
	*Used to could*	*Can could*	*Can will*	*Will can*	*Would should*	*Will would*	*Could can*	*Will might*
	144	135	111	111	81	80	60	59
	*May could*	*Might will*	*Would will*	*Could would*	*Would used to*	*Should could*	*Might ought to*	*Would might*
	53	52	52	41	40	33	32	29
	*Will may*	*Will should*	*May will*	*Can may*	*Will shall*	*Can should*	*Could might*	*Used to would*
	26	23	22	21	21	20	20	20
	*Will could*	*Could should*	*Could will*	*Should will*	*Can might*	*Can would*	*May should*	*May would*
	20	19	16	16	15	14	14	14
	*Should can*	*Might may*	*Should must*	*Can must*	*Will must*	*Could may*	*Would can*	*Might must*
	14	13	12	11	11	10	9	8
	*Must might*	*Should might*	*Used to can*	*Would may*	*May might*	*Must used to*	*Must will*	*Shall should*
	8	8	8	8	7	5	5	5
	*May must*	*May ought to*	*Would must*	*May shall*	*Must could*	*Must would*	*Shall can*	*Shall will*
	4	4	4	3	3	3	3	3
	*Should ought to*	*Can shall*	*May used to*	*Shall must*	*Might shall*	*Might used to*	*Must should*	*Ought to should*
	3	2	2	2	1	1	1	1
	*Shall would*	*Should shall*	*Should used to*	*Will ought to*				
	1	1	1	1

(5) *I* ***might can*** *just come and scoop you we’ll see tho*
  *I* ***might can*** *get a little something*
(6) *Shit* ***might could*** *get real on Friday*
  *I* ***might could*** *go later but no promise bc I’m hauling with Kagen today*
(7) *war hung these alien videos; I want to encounter an alien that* ***should would*** *be dope*
  *If KC actually ends up blowing this lead that* ***should would*** *just be straight up wild*
(8) *I wish I* ***would could*** *do my senior year of high school all over again*.
  *The sad part about me is that I like someone I know I* ***would could*** *never have a chance with*‥
(9) *I* ***will should*** *go to sleep but then again… I won’t*
  *The spurs* ***will should*** *win in 5 against the griz*.
(10) *A call from you* ***would might*** *make the tears stop falling*
  *but I* ***would might*** *accidentally flirt with her*

Two important findings emerge immediately from these results. First, *might could* is not the most frequent DM in the corpus, with its overall frequency only around half that of the most common DM, *might can*. This is very surprising given previous research, which almost always presents *might could* as the most common American DM, while *might can* is often ignored altogether, even though it is the second most common DM in MultiMo. We cannot be certain why this is the case. It may reflect social differences, as we know that the Twitter user base does not match the general population, with greater representation of African Americans and younger Americans, perhaps most notably. It may also reflect a register difference. For example, *I* is the most frequent word in our corpus, whereas in most written registers it is *the*. This finding could also reflect change over time, given that most previous DM data was collected decades ago. It is also important, however, to consider the possibility that previous research has greatly underestimated the general importance of *might can*. We can certainly see how the importance of an individual DM could have been underestimated by fieldworkers, for example, if it was especially common in a community that had not been the focus of fieldwork. We return to this issue below, but for now we highlight that *might can* is surprisingly frequent in our corpus: further large-scale empirical research is necessary to verify if this is the most common form in American English more generally, as well as whether the relative frequency of DMs varies across registers, but *might can* nevertheless appears to be far more common than has previously been acknowledged.

The rankings of some other DMs are also surprising. On the one hand, we find a substantial number of DMs with *will* (e.g. *will can*), *would* (e.g. *would might*), *can* (e.g. *can could*), and *could* (e.g. *could would*) in first position, which are nearly absent in MultiMo. The DMs *must can* and *should would* are also relatively common in our corpus and absent from MultiMo. On the other hand, *used to would* and *should oughta* are not as common in our corpus as in MultiMo, while several DMs starting with *ought to* (*ought to could, ought to will, ought to would*) that occur in MultiMo are absent from our Twitter corpus altogether. Generally, DMs with *may* (e.g. *may can, may could*) and *might* (e.g. *might would, might should*) in first position, as well as *used to could*, are associated with a substantial number of examples in both our corpus and MultiMo. Notably, these results partly align with recent corpus-based research [13], which finds substantial numbers of tokens of *will can* and *could might* but otherwise relatively few of combinations involving *will, would, can*, or *could* in first position (e.g. *would should, would could, can could*), and no tokens of *should would*.

**Fig 1:**
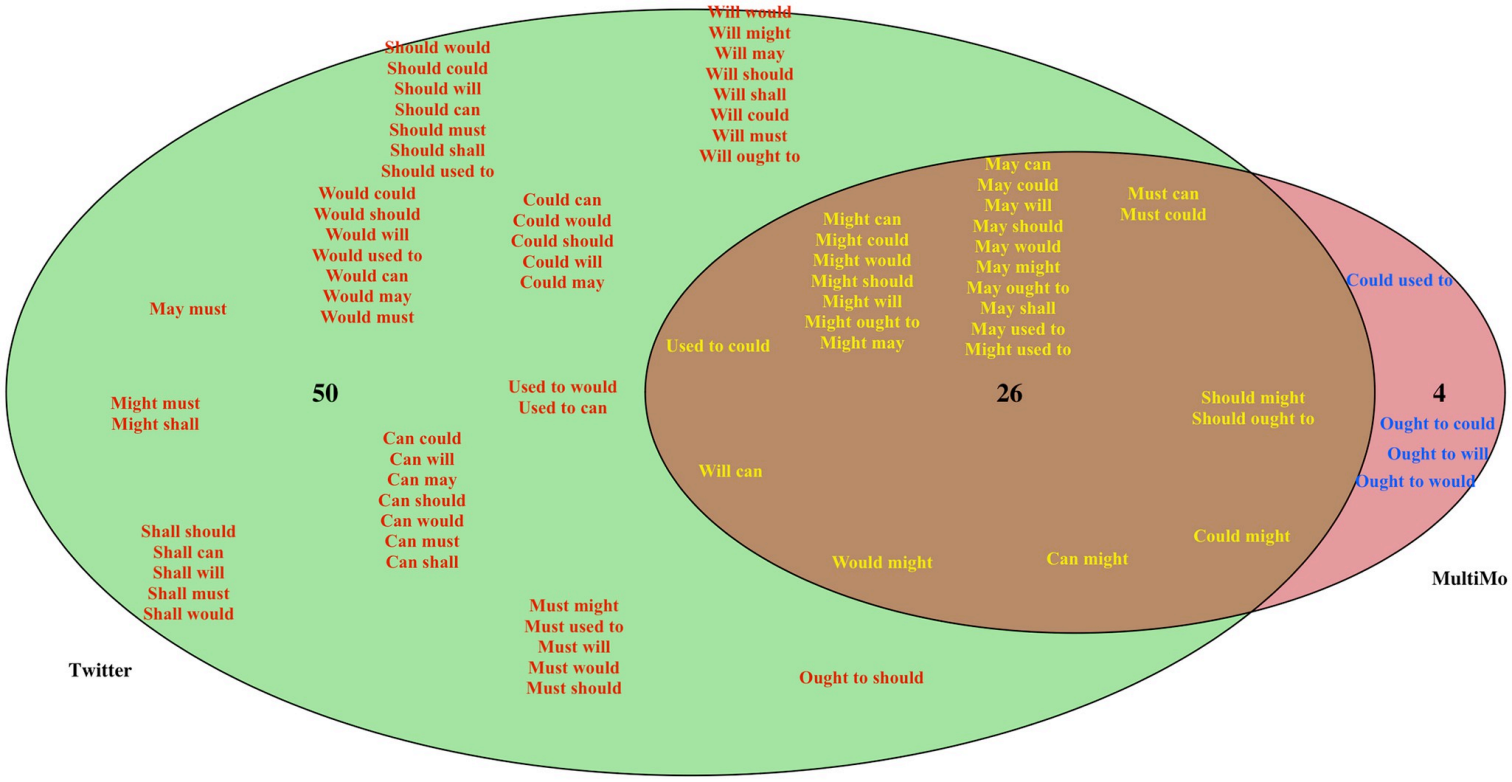
Venn diagram comparing the distribution of DMs in the MultiMo database [46] and the Twitter corpus.

In addition to the unexpected usage of various DMs, our second finding is that the overall diversity of the DMs in our Twitter corpus is greater than has been observed in previous studies, totaling 76 unique combinations out of the possible 110, over twice as many types as found in MultiMo. Although many of these DMs are extremely rare, in general all DMs appear to be used in a purposeful and meaningful way, as validated through manual data cleaning, as discussed in Section 3.2. This surprising result is illustrated in Fig 1, which shows the overlap of DM types between our dataset and MultiMo. Notably, these results broadly align with Coats [13], who finds 37 DM types that are not included in MultiMo.

### 4.2 Regional distribution of double modals

To explore the use of DMs in more detail, we took advantage of the geolocation information in our corpus to map DM use at high resolution across the counties of the contiguous US for the first time. We measured the relative frequency of each DM in each of the 3,075 county sub-corpora by dividing the number of DM tokens in posts from that county by the total number of word tokens in posts from that county, multiplying this value by 1 million to compute rate of use per million words. By normalizing our counts in this way, we controlled for variation in the amount of data available per county, which varies substantially, roughly according to county population. After repeating this process for all 3,075 counties, we then plotted these results using choropleth maps to identify regional patterns in the rate of use of that DM across the US.

**Fig 2:**
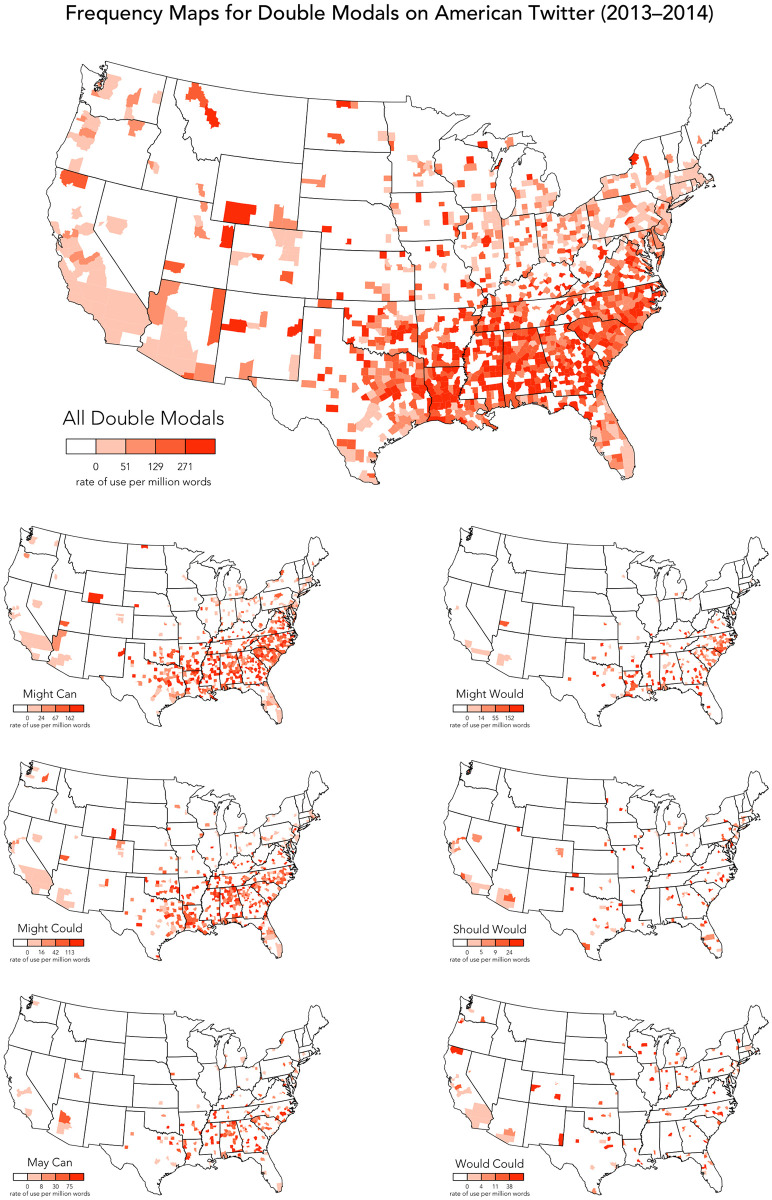
Frequency maps for DMs on American Twitter.

An overall map, showing the combined relative frequencies of all 76 DMs, is presented in Fig 2, along with individual maps for the first six most common DMs. The overall map confirms that DMs are most common in the southeastern US, in a region stretching from Virginia to Texas, including Tennessee, Arkansas, and Oklahoma, although DMs also occur across the rest of the US. It is also notable that the four most common DMs seem to show similar distributions across the South. This southeastern distribution is broadly consistent with previous research on American DMs, which has always focused on the South, but it is notable just how clearly the distribution of DMs aligns with this region (e.g. reflecting the border between the Union and the Confederacy). DMs are also relatively common in states that border the South, especially in Pennsylvania, Ohio, Indiana, and Illinois, although DMs surprisingly tend to be somewhat less common, or at least less widely distributed, in West Virginia, Kentucky, and Missouri. A number of other relatively common and uncommon DMs, as listed in (11), also follow this same general pattern.

(11) **Southeastern DMs**: *might can, might could, may can, might would, must can, might should, used to could, may could, might will, might ought to*
  **Lower frequency**: *used to would, may might, may should, may would, must used to, must will*

**Fig 3:**
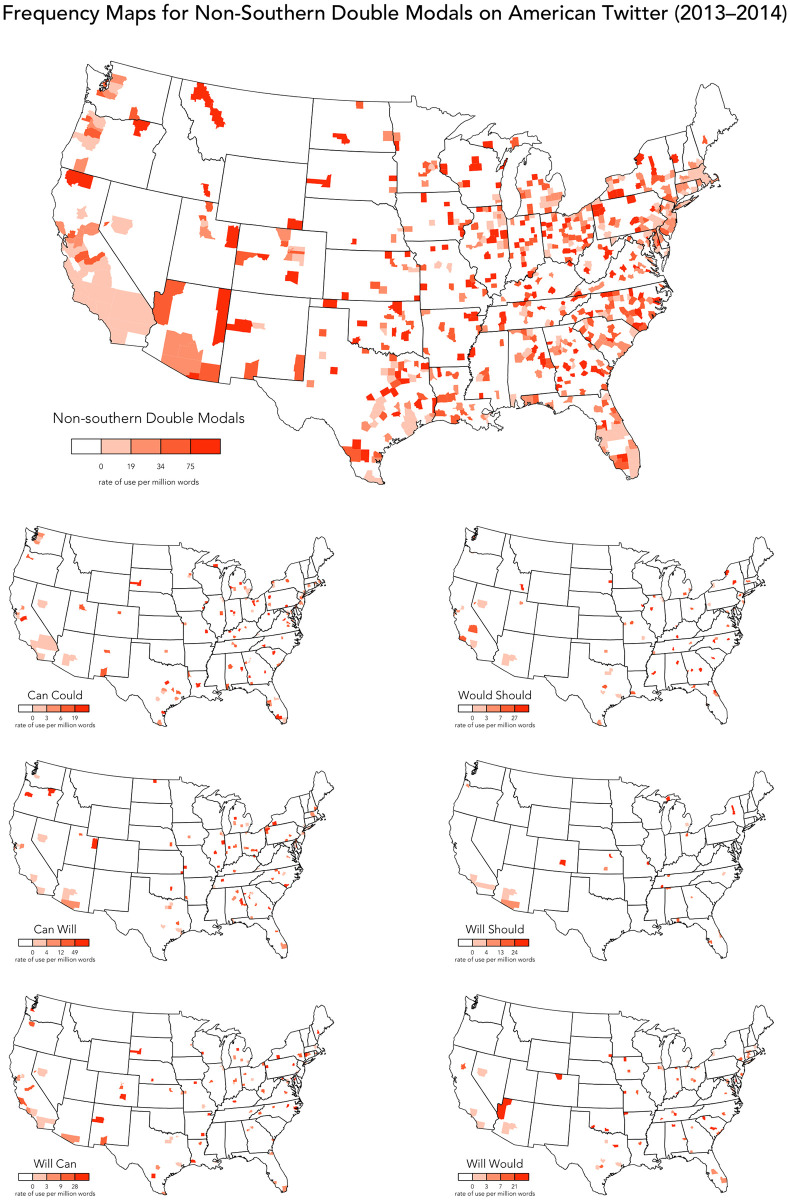
Frequency maps for non-southern DMs on American Twitter.

However, in addition to DMs in the South, our maps also identify a second general pattern: a number of DMs occur across the US, with no discernible regional clustering, including no clear association with the South. These *non-traditional* DMs include *should would* and *would could*, the fifth and sixth most common DMs in our corpus, which are mapped in Fig 2. In fact, over half of the DMs we have identified, as listed in (12), appear to exhibit no regional pattern, although many of these DMs are very uncommon, making it difficult to identify patterns reliably. Six additional examples of these non-traditional DMs are mapped in Fig 3, along with an aggregated map based on the full set of DMs that show no clear regional patterns (as listed in 12), showing that these forms are widely distributed across the US, roughly in line with population density, clearly contrasting with the overall map presented in Fig 2.

(12) **DMs without clear regional patterns**: *should would, would could, can could, can will, will can, would should, will should, will would, could can, will might, would will, could would, would used to, should could, would might, will may*
  **Low-frequency**: *may will, can may, will shall, can should, could might, used to would, will could, could should, could will, should will, can might, can would, may should, may would, should can, might may, should must, can must, will must, could may, would can, might must, must might, should might, used to can, would may, may might, must used to, must will, shall should, may must, may ought to, would must, may shall, must could, must would, shall can, shall will, should ought to, can shall, may used to, shall must, might shall, might used to, must should, ought to should, shall would, should shall, should used to, will ought to*

Our analysis has therefore identified a large group of DMs that are not associated primarily with the South, challenging the view that DMs are exclusively southern forms, in line with the results presented recently in [13]. Before accepting such surprising results, we must question the status of these non-traditional DMs.

At the most basic level, we may wonder if they are valid uses at all. A couple of these combinations could ultimately be typos or results of autofill, although these are likely limited. Firstly, as described in Section 3, each DM token was carefully analyzed by hand to ascertain if they were used meaningfully and purposefully in context for the expression of modal meaning. Through this process, we were able to discard clear errors, and we checked the direct interpretability of the examples in context. Crucially, all DM tokens consist of two modal verbs in a single tensed clause. Secondly, the long tail of low-frequency DMs observed in Table 3 is important evidence for the validity of the results. Notably, the distribution does not show a clear inflection point or cut-off, after which we would only see sporadic use of invalid forms. Furthermore, Coats’s previous study of DMs, which uses manually checked YouTube video excerpts with detailed recordings of oral speech [13], broadly supports the diversity of DMs found in our study, also identifying a long tail of authentic, though very infrequent DMs. In sum, although we find low frequency and considerable variability in the use of DMs in our corpus, including many previously unattested combinations, these forms, at least in the vast majority of cases, are used in a way that is both purposeful and meaningful.

For example, one noteworthy tweet in our data is by Statik Selektah (@StatikSelekt), a well-known white hip-hop producer from Boston. He is the author of the only tweet in our corpus containing *might shall*, which he posted in response to a private tweet, in what seems like a humorous context, given the use of a laughter marker and the use of formal *shall* in a DM, an inherently informal construction.

(13) a. *I might shall come. lol*.

Even in this case, however, it seems the user is well aware of the existence of the DM *might will*, a relatively well-established traditional DM, which may have been mentioned in the original tweet, which is not accessible. Although some tokens of non-traditional DMs that we observed in this study must be unintentional typographic errors, as is likely for the analysis of any feature in a corpus, there are many examples of these forms overall, all of which were hand checked and judged to be valid in context, as illustrated in previous examples. These non-traditional DMs therefore appear to be a very real although very rare feature of American English—one that has not generally been acknowledged and that is not easily explained given current theories of DMs.

## 5 Theoretical discussion

### 5.1 Semantic constraints on double modal formation

Because of the lack of information about the inventory of DMs and their relative frequencies, it has been difficult to identify how modal verbs can combine to form DMs in American English. The descriptive results presented in Section 4.1, however, provide us with an empirical basis for assessing the semantic constraints on DM formation in detail.

**Fig 4:**
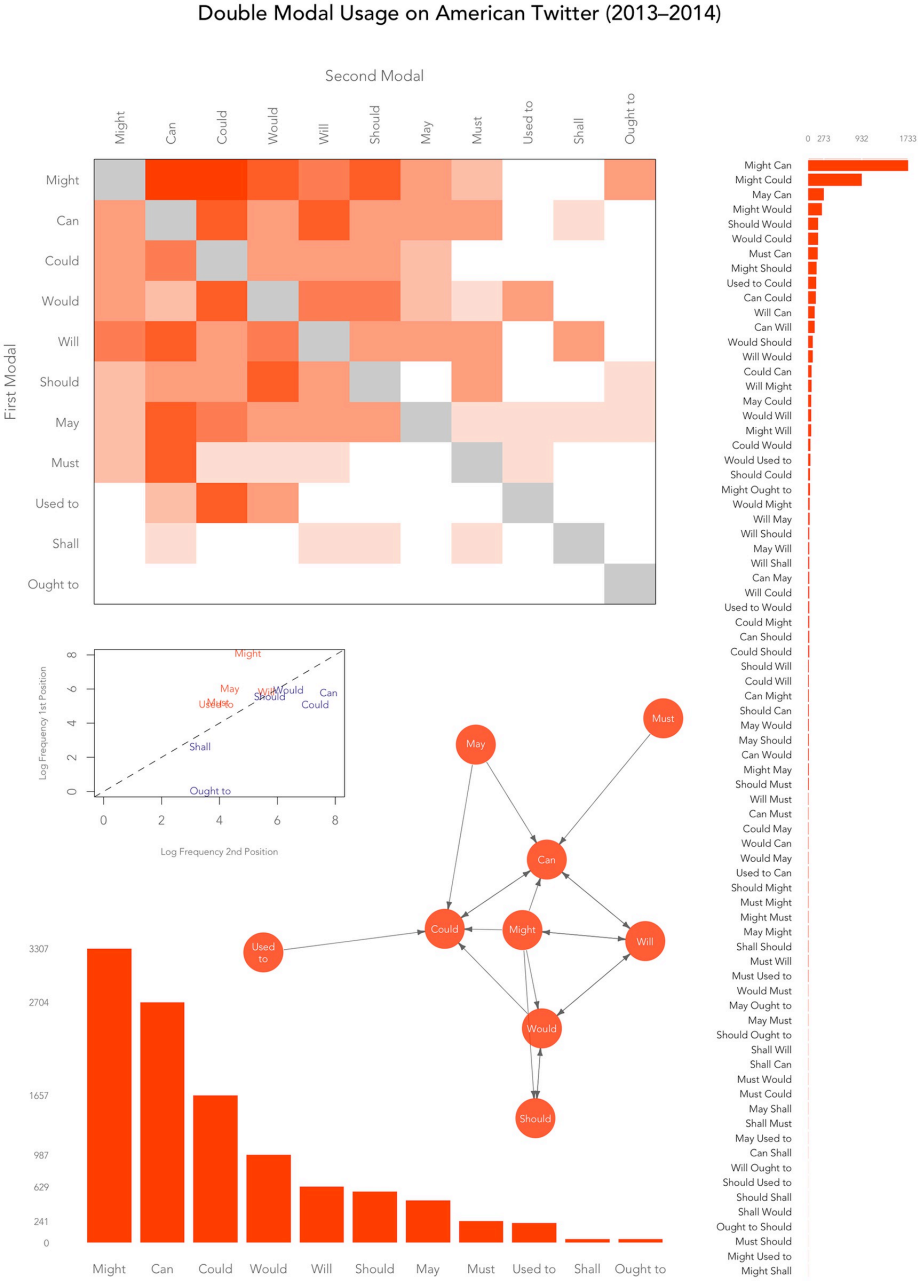
Visualizations of DMs in the Twitter corpus (clockwise from top): heatmap by modal position, histogram for DMs, Markov chain for modal position, histogram for individual modals in DMs, and DM scatter plot by modal position.

Although the frequency of DM types is highly skewed toward a small number of relatively high frequency forms, as can be clearly seen in the DM histogram on the right side of Fig 4, there is a great diversity of DM types overall, which incorporate a range of different modal verbs, as illustrated by the histogram for individual modals at the bottom of Fig 4. This is shown more clearly in the heat map in Fig 4, which plots which modal combinations are most common. For example, *might can* and *might could* are the two most common DMs in our corpus and these combinations are therefore highlighted in dark shades. Overall, this heat map clearly shows the complexity of DMs in American English.

The heatmap also helps us better see the prominence of certain modals in first (*might*, *may*, *will*, *would*, *can*) and second (*can*, *could*, *will*, *would*) position. Alternatively, it highlights that *used to* and *ought to* are generally rare in either position, while isolating *used to could* as a common DM with an uncommon first modal. Other common DMs that stand out as being characterized by an uncommon placement of a modal include *must can*, *should would*, and *might should*. The scatter plot in Fig 4 distills this information by plotting each modal based on its overall logged frequencies in first and second position. The diagonal line distinguishes between modals that are prominent in first position (above the line, in red), including *might*, *may*, *must*, and *used to*, and modals that are prominent in second position (below the line, in blue), including *can* and *could*. Alternatively, *will*, *would*, and *should* occur in first and second position at a similar rate.

The relationships between individual modals based on their co-occurrence patterns is also visualized using a Markov chain in Fig 4. This plot illustrates how modal verbs tend to combine to form DMs, with directed links between the modal verb nodes representing the most common combinations of modal verbs in first and second position of DMs in our corpus. Notably, this Markov chain only shows links between modals with more than 50 total tokens across all DM types so as to allow us to focus on the most important relationships between individual modal verbs, resulting in the modals *shall* and *ought to* being excluded. Specifically, the Markov chain shows that *might* combines with *can* and *could*, but not the other way round, whereas *will* occurs commonly with three modals (*can*, *might*, *would*), in both first and second position. As suggested in previous visualizations, *might*, *could*, and *can* are clearly at the core of this phenomenon, with 5, 6, and 7 links in and out respectively. A more surprising result is that *will* and *would* are also very common, each with 6 links in and out.

Crucially, these results show that, although there is great variability in the relative frequency of different DM types, there are no absolute constraints on the formation of DM types: constraints on DM formation is also probabilistic. This is of considerable importance with respect to theories of DM structure, which have generally assumed a highly constrained DM system in American English, where the DM system is governed by specific positions assigned to specific modals. Indeed, given these results, it seems likely that given a sufficiently large corpus we would observe all 110 of the modal combinations for which we searched.

To better understand these probabilistic semantic constraints on DM formation, we applied a framework based on the classic distinctions delineated by Coates and Palmer [65–67], especially the distinction between *epistemic* and *root*. Epistemic modality expresses a speaker’s degree of confidence in the likelihood of a proposition being true, while root modality expresses a speaker’s attitude regarding the possibility of the subject being involved in an event (e.g. desirability, capacity, or obligation). Root modality is further subdivided into *deontic* modality, which refers to a source that is external to the speaker (e.g. a source of authority), and *dynamic* modality, which refers to the speaker as a source (e.g. a source of capacity). Importantly, many individual modals can convey either epistemic or root meanings. We also draw on the distinction between *possibility* and *necessity* modals, simplifying Biber and colleagues’ [68] classification by treating prediction as a form of epistemic necessity and volition as a form of root/dynamic necessity. The degree to which modal categories such as Epistemic and Root are semantic or pragmatic in nature is an ongoing discussion in modality studies [69]. For the purpose of this study we consider these categories as primarily semantic, acknowledging a finer definition at the semantics-pragmatics interface is ultimately desirable.

Following this system, we first manually annotated a set of DM tokens consisting of a random sample of 50 tokens from each of the 10 most frequent DM types. Notably, the meaning of a modal occurring in a specific DM type can vary depending on context. Coding each token therefore required taking into account the immediate context of the Tweet in which the DM was used, which is restricted, given the shortness of posts. Although it is, of course, impossible to recover the precise intent of the author of each Tweet, there are features that generally allow us to recognize modality type and interpret the likely intended meaning as native speakers of English. Most importantly, these features include examining the subject and the main verb type of the sentence in which the DM occurs to see whether the modal scopes over an entire proposition or the participant specifically, as well as punctuation marks, interjections, and emojis that convey speaker attitudes and judgments about propositions and events. We present 10 examples where we have annotated the modality type of each modal in various DMs in (14).

(14) a. *lol hush. I might surprise people!! I* ***might can*** *dunk on emmmmmm lol* (Epistemic–Root/dynamic, Possibility–Possibility)
   *we live in Miami that dream* ***might can*** *happen* 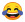 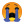 (Epistemic–Epistemic, Possibility–Possibility)
  b. *haha oh you think so? you* ***might could*** *start by texting back!* (Epistemic–Root/deontic, Possibility–Possibility)
   *Shit* ***might could*** *get real on Friday* 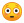 (Epistemic–Epistemic, Possibility–Possibility)
  c. *can you email me the details I* ***may can*** *join you two :)* (Epistemic–Root/deontic, Possibility–Possibility)
  d. *You should have said no and baby you* ***might would*** *still have me* (Epistemic–Epistemic, Possibility–Necessity)
   *Wasn’t for work I* ***might would*** *teach yal young whipper snappers something lol* (Epistemic–Root/dynamic, Possibility–Necessity)
  e. *Even tho I ain’t feeling you. It* ***should would*** *be good tonight* (Epistemic–Epistemic, Necessity–Necessity)
   *You* ***should would*** *know if its a fake account (common sense) but nooooo with your thirsty ass to disparate for a female* ‥ (Root/deontic–Epistemic, Necessity–Necessity)
  f. *I wish I* ***would could*** *do my senior year of high school all over again*. (Epistemic–Root/deontic, Necessity–Possibility)
   The author appears to be epistemically asserting (*would*) their capacity (*could*) to carry out the event.
   *I wonder what it* ***would could*** *be like‥ to still be playing‥ to chase the dream*. (Epistemic–Epistemic, Possibility–Possibility)
  g. *Prynce* ***must can*** *tell I’m sad…. He’s moping around right with me. He won’t even eat‥*. (Epistemic–Root/dynamic, Necessity–Possibility)
   *If you talking shit you* ***must can*** *back it up. If not your gonna get beat up* 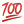 (Root–Root, Necessity–Possibility)
  h. *I just cussed because of flappy bird. I* ***might should*** *delete this app now* (Epistemic–Root/deontic, Possibility–Necessity)
  i. *I* ***used to could*** *sleep till 12-1pm…nope not anymore! It’s official; I’m getting old* 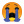 (Epistemic–Root/dynamic, Necessity–Possibility)
  j. *The worst thing you* ***can could*** *do is message me when my team loses… You’ll get deleted real quick*. (Epistemic–Epistemic, Possibility–Possibility)
   *I wish I had my car already so I* ***can could*** *just leave*. (Root–Root, Possibility–Possibility)

Based on these annotations, we computed the proportions of the four different possible Epistemic and Root orderings for each of the 10 most common DMs, as presented in Table 4. Overall, although we can clearly see that semantic patterns in modal combinations is probabilistic, we do find the general ordering pattern of Epistemic > Root predicted by the literature for these most common DMs [8, 25, 26]: the typical combination is Epistemic–Root (57% of all tokens across all 10 DM types), which is also the most frequent ordering for *might can, might could, may can, would could, must can, might should*, and *used to could*. Notably, Epistemic–Epistemic ordering is also relatively common (28%), and is the most frequent ordering for *should would* and *might would*. Root–Root ordering is considerably rarer (11%), only attested for the DMs *must can* and *can could*, for which it is the most common ordering. Finally, Root–Epistemic ordering is most uncommon (4%), but is not completely absent, attested a substantial number of times for *should would*, and rarely for *must can*, demonstrating that not even the Epistemic–Root constraint is categorical.

**Table 4:** Proportions of semantic ordering of DMs in random samples for the top 10 DMs (n = 50).

DM	Epistemic First		Root First	
	E–E	E–R	R–R	R–E
*Might Can*	0.2	0.8	0	0
*Might Could*	0.3	0.7	0	0
*May Can*	0.14	0.86	0	0
*Might Would*	0.7	0.3	0	0
*Should Would*	0.64	0.06	0	0.3
*Would Could*	0.32	0.68	0	0
*Must Can*	0	0.76	0.22	0.02
*Might Should*	0.04	0.86	0	0
*Used to Could*	0.02	0.98	0	0
*Can Could*	0.2	0	0.8	0
**Average (rounded)**	0.28	0.57	0.11	0.04

Two important additional patterns emerge from examining the top 10 DM types together. First, possibility modals are clearly more common overall, including the top three most common modals (*might*, *can*, *could*). DMs therefore seem to be primarily used to specify and emphasize possibility modality. This finding corroborates suggestions made in the literature, especially with respect to hedging and mitigation [10, 11, 19]. However, necessity modals do occur in the top 10 DMs, including *would*, *will*, and *should*. Again, this preference is thus probabilistic. Second, our results allow us to evaluate the claim that DMs generally display a *matched-tense* constraint [24], where the two modals are either both *present* or both *preterite* forms (i.e. *may/might, can/could, will/would*, and *shall/should*), where the preterite is considered a tentative version of the present form, conveying additional irrealis as opposed to indicative meaning [66]. Our results show that the most frequent DMs are generally characterized by matched tense, most commonly involving preterite forms, which provides additional evidence for the function of DMs as mitigating tools. For example, out of the 10 most frequent DMs, 8 show tense agreement, 6 of which are preterite. However, the very large number of DM types observed in this study, including *might can*, the most common DM type in corpus, demonstrates that mixed-tense DMs are common as well, showing once again that the matched-tensed constraint can only be described as probabilistic.

In addition, we coded a second set of DM tokens consisting of all tokens for all DM types with 5 or less tokens. Unsurprisingly, we find that the constraints on DM formation described above are more likely to be violated for less common DMs. Table 5 reports the ordering of DMs for the 59 tokens of the 23 DM types that occur 5 times or less in the corpus, showing that the four possible orderings account for a roughly equal proportion of tokens, as illustrated in the examples presented in (15). DM combinations appear to be essentially unconstrained at the bottom of our observed frequency distribution.

**Table 5:** Proportions of semantic orderings for the 23 DMs with 5 tokens or less (*n* = 59).

DM	Epistemic First		Root First	
	E–E	E–R	R–R	R–E
*23 least common DMs*	0.32	0.25	0.25	0.18

(15) a. *Well by the way things are going so far. We **may should** predict a cold summer?* (Epistemic–Root/deontic)
  b. *I* ***used to would*** *do anything to feel loved; but now I’ll do anything for y’all to not even be cordial*. (Epistemic–Epistemic)
  c. *All that retweetin she doing she* ***must will*** *DM me* 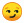 (Epistemic–Epistemic)
  d. *i* ***must used to*** *really tick you off then. my attitude used to be awful!* (Epistemic–Epistemic)
  e. *If you didn’t have to sell your soul; I* ***may would*** *join a club* (Epistemic–Root/dynamic)
  f. *hahah I’m never having a party again 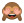 but we* ***can shall*** *hangout :)* (Root/deontic–Root/deontic, Possibility–Necessity)
  g. *Yo that LG G-Flex* ***must might*** *be my next phone*. (Root/deontic–Epistemic, Necessity–Possibility)
  h. *Jared is the one who wears scarves. Thus I* ***can must*** *conclude that is Jared’s scarf. Hey; isn’t trading scarves LIKE TRADING RINGS?? HMMMM?* (Root/deontic–Epistemic, Possibility–Necessity)
  i. *food I got! It’s what I want to eat I don’t have!”and I* ***could may*** *get it* (Root/dynamic–Epistemic, Possibility–Possibility)
  j. *Maybe I* ***should might*** *make a lot of people happier*‥ (Root/deontic–Epistemic, Necessity–Possibility)

Overall, we therefore find that DM combinations appear to be far less constrained than has been assumed, in line with recent corpus-based research on spoken English [13]. Although the most frequent DMs in our corpus are characterized by probabilistic patterns in the semantic types of modals that tend to combine, in general any DM combination appears to be possible. The relative frequencies of DM types therefore seems to reflect the pressure of language use as opposed to any absolute underlying constraints on which modals can be combined. These pressures likely include speakers taking advantage of DMs to express fine-grained differences in modality, with certain meanings being more or less useful and therefore certain DMs more or less common, although this variation may also reflect social meaning, as we discuss in the next section.

### 5.2 Sources of dialect variation in double modal use

As described in Section 2.2, we have identified two basic regional patterns in the relative frequencies of individual DMs: many of the most frequent and familiar *traditional* DMs are used primarily in the South, as we would expect, but there is also a large number of *non-traditional* DMs that occur across the US without any apparent regional pattern. We return to these non-traditional DMs in the next section when we discuss the origins of DMs, but first we zoom in on the South to assess theories of regional and social dialect variation of traditional DM use.

**Fig 5:**
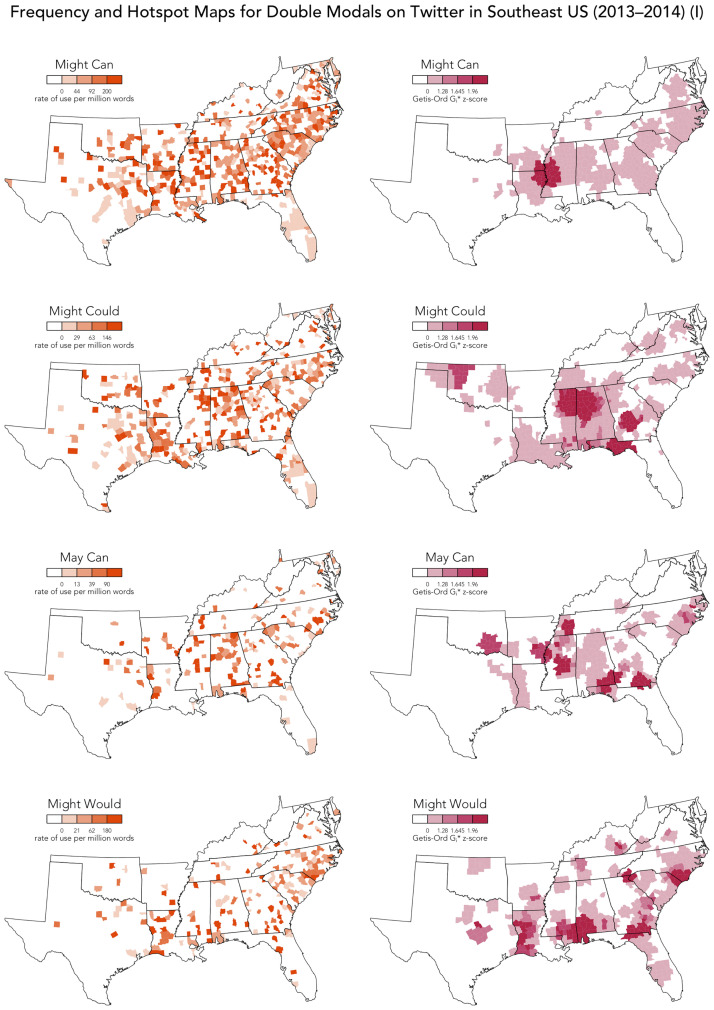
Frequency and hostpot maps for DMs on Twitter in Southeast US.

In this section, we focus on the use of the 10 most frequent traditional DMs that predominate in our corpus: *might can, might could, may can, might would, must can, might should, used to could, may could, might will*, and *might ought to*. Because these DMs are relatively frequent on Twitter, their usage can be mapped in high resolution across the South, allowing us to analyze their regional distribution in greater detail than has previously been possible, including conducting a local spatial autocorrelation Getis-Ord Gi* analysis (using a 25 nearest neighbor spatial weights matrix). Local spatial autocorrelation analysis is a method commonly used in dialectometry to isolate the underlying regional patterns from noisy dialect maps [70]. Specifically, given a variable measured over a series of locations, a local spatial autocorrelation analysis returns a *z*-score for each location quantifying the degree to which it is part of a high (positive) or low (negative) value cluster. These values are then mapped to identify geographical hotspots in the value of the variable. The relative frequency maps and the hotspot maps for the four most common DMs are presented side-by-side in Fig 5, allowing for the effects of the spatial autocorrelation analysis to be assessed. Based on these maps, we can better appreciate how these DMs are used across this region.

In terms of theories of dialect variation, what is immediately notable is the relatively low rates of usage of DMs on Twitter in Appalachia (i.e. in West Virginia, western Virginia and North Carolina, and eastern Kentucky and Tennessee). Rather, broadly speaking, we find that DMs are much more common on Twitter in the Deep South (i.e. in South Carolina, Georgia, Alabama, Mississippi, and Louisiana). This pattern can also be appreciated in the overall map of DMs presented at the top of Fig 2, which is dominated by these most frequent traditional DMs.

This spatial distribution is very surprising given previous claims in dialectology and sociolinguistics that DMs are most strongly associated with dialects of American English spoken in Appalachia [22]. It may be that our data source largely excludes DM users from Appalachia, which is both a relatively rural area and impoverished area. It is notable, however, that we have considerable data from across the South, including Appalachia. Our maps are also controlled for variation in sample size, and we do find relatively high usage of DMs in relatively rural and impoverished areas in the Deep South. Notably, the Deep South has a very different demographic makeup than the Upper South, being characterized especially by much larger African American populations, reflecting the history of slavery in this region. Furthermore, looking at our maps, it appears that DMs are used especially frequently on Twitter in parts of the Deep South with especially larger proportions of African Americans, in line with the findings of the LAGS dialect survey (see Table 2), which focused on the Deep South, and found generally higher usage of DMs among African Americans (aside from *might could*).

**Fig 6:**
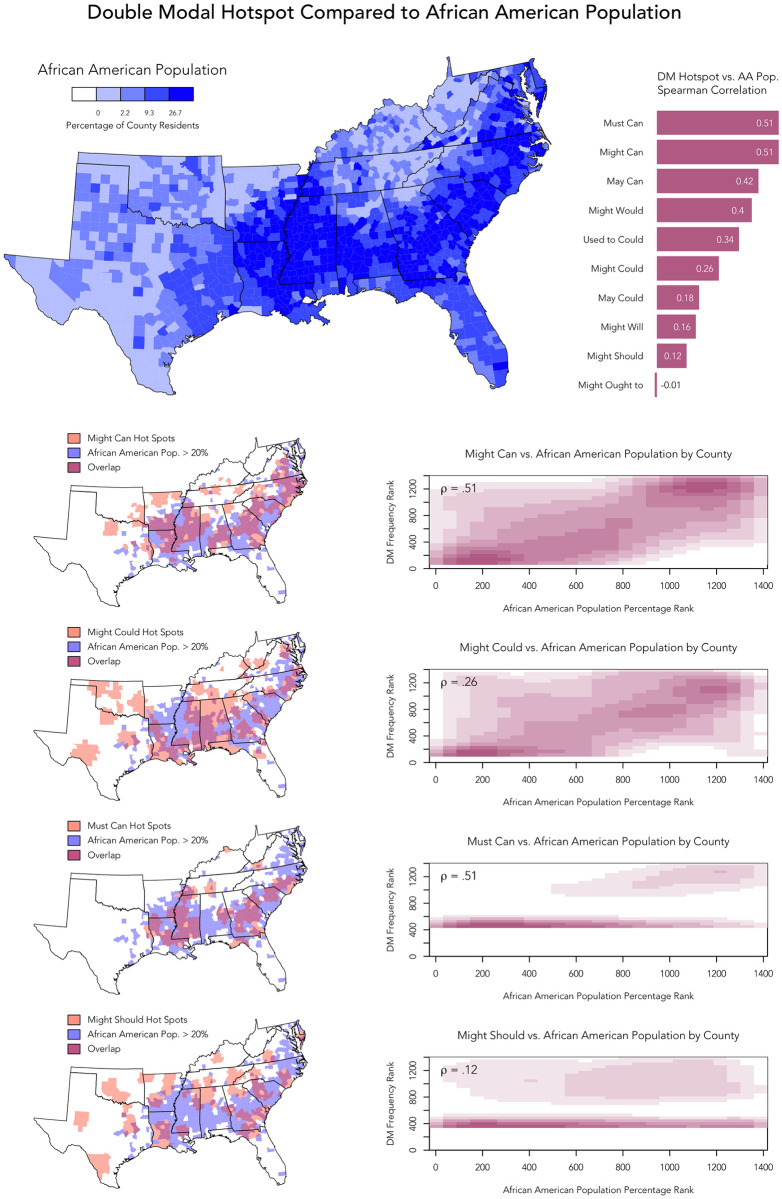
DM hotspots on Twitter compared to African American population in the Southeast US.

To explore the relationship between DM usage and AAL in the South in more detail, we compared the percentage of African Americans (2010 US Census) to the local spatial autocorrelation maps for each of the 10 DMs by calculating a Spearman correlation coefficient between these two maps across southeastern counties, where a higher value represents a greater similarity in the two maps. We use a rank-based correlation coefficient because the distribution of these two variables differ substantially. The results of this analysis are presented in Fig 6, which also includes a map of African American population in the region for reference, as well as maps highlighting the overlap between areas of high African American population and high DM usage. In addition, we include a kernel-density-estimation scatter plot for each DM across all counties showing the relation between the ranked relative frequency of that DM compared to the ranked percentage of African Americans by county. We plot ranked values because we use a ranked-correlation coefficient, and we plot these values using kernel density estimation to allow for the relationship to be appreciated despite the large number of overlapping observations.

Overall, correlations vary from -.01 to .51, as presented in the histogram and as reflected in the scatter plots in Fig 6, where a larger positive correlation represents a stronger association with counties with relatively high proportions of African Americans. Overall, we therefore find that the traditional DMs tend to be associated with AAL: the relative frequency maps for 9 out these 10 most frequent traditional DMs show positive correlations with the distribution of African American populations in the South, with a number of DMs, especially *must can, might can, may can, and might would*, showing especially strong correlations (*ρ*≥.4). In general, it therefore appears that DMs with *can* in second position are especially strongly associated with AAL.

More specifically, we find that the regional distribution of *might can* on Twitter—which, as we have noted, is surprisingly the most common DM in our corpus—is one of the two most strongly correlated to the distribution of African Americans in the South (*ρ* = .51), suggesting an especially strong link to AAL. Alternatively, *might could*, the second most frequent DM in our corpus—which has always been assumed to be the most common DM—exhibits a much weaker correlation (*ρ* = .26). This difference can be seen especially clearly in the overlap maps for these two DMs: while hotspots for *might can* largely occur within areas of high African American population, hotspots for *might could* largely occur outside these areas, especially in the upper thirds of Georgia, Alabama, and Mississippi, as well as in Tennessee and Oklahoma—regions with substantially smaller African American populations. The other 8 DMs show similar patterns, depending on the strength of this correlation. For example, overlap maps are also provided for *must can*, which also appears to be strongly associated with AAL (*ρ* = .51), and *might should*, which appears to be less strongly associated with areas with large African American populations (*ρ* = .12).

The finding that there is variation in the types of DM commonly used by Whites and African Americans in the South, at least on Twitter, is a new and unexpected result. Previous research on DMs has identified only limited social and regional variation. Our results not only support the claim that DMs are generally more widespread among African Americans [15, 71], but they imply that African Americans and White Americans in the South use DMs in quantitatively different ways, favoring different DM types. The degree to which we can speak of two different DM systems is unclear, but it does seem there is at least a continuum of DM types in the South, with DMs more strongly associated with AAL, like *might can*, at one extreme, and forms that are more neutral, and thus arguably more associated with general white Southern English, like *might should* and *might ought to*, at the other.

In other words, DM usage appears to have relatively clear social meaning: it is not simply the case that using traditional DMs in general indexes being Southern, although this appears to be true to an extent, but additionally, within the South, variation in the specific DM types that are used indexes different communities, with the use of certain DMs being especially strongly associated with African Americans.

In terms of sociolinguistic variation, the use of *might can* and *might could* is potentially especially informative, as these are (1) the two most frequent DMs in the corpus, (2) two forms that vary substantially in terms of their association with AAL, and (3) two forms that have relatively similar meanings, both exhibiting Epistemic–Root and Epistemic–Epistemic modality, with their constituent modals both expressing possibility. Consequently, these two DMs appear, in terms of their basic referential meaning, to often be interchangeable in use, with perhaps the main difference in their meaning being that *could* weakens the force of the expression somewhat more than *can* [8], as illustrated in (16). The primary difference between these DMs, in many cases at least, therefore appears to be their *social meaning*, as opposed to their *referential meaning*, with *might can* appearing to index a more African American identity at least online.

(16) a. (i) *if y’all aren’t doing anything we* [***might can*** *go tonight] 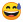 depending on what I am doing*
   (ii) *Glenn* [***might could*** *go tonight]* …
  b. (i) *wassup then ?we [**might can** do that]*
   (ii) *I* [***might could*** *do that]!!!*
  c. (i) *what size?? I [**might can** help ya out]!*
   (ii) *a GPS* [***might could*** *help ya out]* 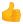  d. (i) *well we [**might can** work something out]!*
   (ii) *alright lol yeah I’m in Pennsylvania we* [***might could*** *work something out] I’ll have to see your work*.

Crucially, this hypothesis also offers an explanation for the surprisingly high frequency of *might can* in our corpus compared to *might could*, which has always been assumed to be the most common DM: it appears that *might can*, as well as other relatively common DMs, are used by a population that has not been adequately represented in previous research. Because research in American dialectology has largely been based on fieldwork carried out by white researchers, who often focused on informants from white communities, the amount of variation in DMs may have been underestimated, especially those DMs that are used primarily by African Americans. This result also likely explains the relatively little DM use in predominantly African American areas of the Deep South identified in recent corpus-based research [13], although it is unclear how exactly data was sampled for that study.

### 5.3 Possible African American origins of double modals

Finally, we believe our findings may have important implications for our understanding of the origins of DMs in American English. As we discussed in Section 2.3, the dominant theory is that DMs were brought to the US by Scots-Irish immigrants, who primarily settled in Appalachia. We find, however, that traditional DMs are relatively uncommon in Appalachia, and the Upper South more generally, and more strongly associated with the Deep South—a result that is broadly in line with Coats’s recent corpus-based study of spoken language collected from YouTube [13]. More specifically, as we argue in Section 5.2, it appears that traditional DMs are used especially frequently by African Americans in the Deep South, at least on Twitter. Even *used to could*, which is relatively common in our corpus, and which is a typical DM in Scots [33, 38], does not show a clear Appalachian distribution, being more common in the Deep South. In addition, we find that many DMs that have been found in the British Isles, such as *must can* and *will can*, are not especially common in our corpus, while more common DMs in our corpus, such as *may can* and *must would*, are not well attested in the British Isles.

These findings clearly challenge traditional theories of DMs origins, as this is not the picture of DMs we would expect to find if DMs had originally diffused outwards from Scots-Irish settlers in Appalachia. That is not to say that the DM system we observe in the South could not have developed in this way. For example, a DM system could have passed from Scots-Irish immigrants in Appalachia, who were often relatively poor and isolated, to more well-established Whites, including slave-holders, in the Deep South, who would have often come from English backgrounds [72], and who would have therefore spoken more prestigious dialects of English that did not contain DMs, and then from there spread to African Americans, including slaves, with both the DM types in this system and their social meaning changing over time, eventually becoming especially common in the language of African Americans. Although this scenario seems possible, it also seems highly complex, which should encourage linguists to formulate and consider simpler explanations for the origins of DMs in American English.

Although modern Twitter data clearly cannot conclusively resolve a historical linguistic debate, we believe our results offer more direct support for an alternative and ultimately simpler theory of DM origins in American English, which has been acknowledged but always been dismissed in the literature: the traditional DM system of Southern American English is an American innovation, specifically of AAL.

In other words, we believe our study provides empirical evidence for the African American origins of DMs. From this source, DMs could have then spread out to White Americans in the South, perhaps eventually encountering different and more isolated DMs as used by Scots-Irish settlers in Appalachia, as well as potentially Gullah speakers in the Sea Islands. As they spread, perhaps due to the fine grained differences in modality they allow speakers to express, some of these DMs could have lost their original social meaning, becoming less strongly associated with African American communities over time, as they were increasingly used by White Americans. Crucially, this would not be an unattested occurrence: at least in modern times, the adoption of AAL innovations by speakers of general American English has often been observed, including in this very corpus [58].

We want to stress, however, that we do not believe this is conclusive evidence in support of this *African American theory of DM origins*, or against the standard *Scots-Irish theory of DM origins*. Rather, we simply believe this African American explanation should be seriously considered as a possible explanation, as opposed to being dismissed outright, as it has so many times in the past. After analyzing an unprecedentedly large number of DM tokens in a corpus of natural language—without relying on our own intuitions and social networks to collect data—we believe the African American origins of the Southern DM system seem far more likely than has previously been assumed, and should be considered to be a plausible explanation for the origins of DMs in American English, in addition to the standard theory that they were imported to America by speakers from Scots-Irish backgrounds.

Of course, if American DMs did originate in AAL, we do not how or why this first system emerged among African American speakers. Indeed, the ultimate origins of DMs in Scots are also unclear. We believe, however, that the non-traditional DMs that we have observed in this study—many of which have never been attested in traditional research and that we find occur across the US without any apparent regional pattern—may help us begin to understand how the southern DM system may have emerged in American English. Crucially, we do not believe the non-traditional DMs we have observed spread via contact with speakers of traditional DMs, as these DMs are not part of the Southern DM system. In addition, it is notable that non-traditional DMs appear to be less constrained by the structural and semantic factors discussed in Section 5.2. For example, the presence of *will, would, can, could*, and *should* in first position is especially striking, while *should would*, the most common of the non-traditional DMs, is characterized by relatively frequent Root–Epistemic meanings. Furthermore, given the lack of regional patterns in these non-traditional DMs, we do not believe they are dialect forms that are associated with specific communities.

Rather, our results suggest that the great diversity of non-traditional DMs observed in this study is evidence there is a *productive* process of DM formation in General American English, independent of the Southern DM system. In other words, in general, it seems that American English licenses DM constructions, without absolute semantic or syntactic restriction, allowing speakers to produce, albeit extremely rarely, novel DM combinations on the fly to suit their specific communicative needs, especially the fine-grained expression of epistemic and root modality.

Our identification of non-traditional DMs implies that these same resources would have likely been available to African Americans in the South. If this assumption is true, the question then is not where did DMs come from, by how did this highly complex specific DM system emerge? Once again, we can only speculate, but one possibility, especially in light of the social patterns discussed in Section 5.2, is that they developed as a way to mark social meaning by drawing on a grammatical but highly marked construction. Another possibility is that this system may have developed as part of the emergence of AAL more generally. In particular, it is notable that DMs are attested if not pervasive across many different English-based creoles and pidgins around the world [3], including Guyanese Creole, Hawaiian Creole, Jamaican Creole, Nigerian Pidgin, Sranan, and Saramaccan, as well as Gullah in the US [15, 71]. The presence of DMs in American English has always appeared to be an exception to this pattern, but there has been a long-standing debate on the creole origins of AAL [73–75]. Given our results, it may therefore be possible that the DM system in AAL arose as part of a creolization process.

These theories of DM origins in American English, and in the English language more generally, are highly speculative, and require further research, although, given the lack of a historical record, we suspect these debates will never be resolved. We believe, however, that the results presented in this study, the most extensive empirical analysis of DM usage that has ever been conducted, challenges standard assumptions about DM origins, and demands that we consider new hypotheses about how these constructions first emerged, including their possible origins in AAL.

## 6 Conclusion

In this paper, we have reported the results of a detailed analysis of DM use in a multi-billion word corpus of geolocated American Twitter data collected between 2013 and 2014. Given their rarity, DMs have always been difficult to study, leading to a limited number of tokens of DMs having been analyzed in the literature, generally collected through forms of elicitation. Access to such a large and informal corpus of natural language has therefore provided us with a unique opportunity to work with a much more meaningful sample of DM tokens, allowing us to assess theories about the structure, use, and origins of DMs in American English from a new and privileged perspective.

First, we believe our findings provide clear evidence that the diversity of DMs in American English is far more complex than has generally been assumed. Out of the 110 possible DM combinations for which we searched, we identified 76 different types on Twitter; presumably, if we had access to considerably more data, we would have identified many additional types, perhaps eventually all possible combinations. Ultimately, it therefore appears that DM formation is unconstrained, in the sense that no particular combination of modals appears to be impossible. As we have shown, there are nevertheless patterns in how modals tend to combine to form DMs. For example, DMs in American English have a preference for *may* and *might* in first position and *can* and *could* in second position, as well as for tense-matched modals and Epistemic–Root orderings. But crucially our findings show that such semantic constraints on DM formation can only be expressed probabilistically, which implies that these patterns are a product of the pressures of language use. We believe this result is evidence that DMs are a productive construction in American English, being formed without absolute constraints, albeit with incredible infrequency, so as to express fine-grained distinctions in modality.

Second, we believe our findings provide clear evidence that the social and regional factors underlying dialect variation in DM use in American English are also far more complex than has generally been assumed, both in the South, the traditional area of DM use, and across the US more generally. In the South, we found that traditional DMs are most strongly associated on Twitter with regions that have especially large African American populations, implying that DMs are especially strongly associated with AAL. We also found that some DMs are more strongly associated with AAL, implying the existence of complex patterns of social meaning in how these forms are used. This is a surprising result given that most research has focused on DMs in the language of White Americans, especially in Appalachia, where we in fact found relatively low use of DMs overall. This pattern also likely explains another surprising finding: despite *all* previous research, we found that *might can* as opposed to *might could* is by far the most common DM in our corpus, which is also one of the two DMs that appears to be most strongly associated with AAL. Given that we have only analyzed one register of American English, it is unclear which of these DMs is more common in American English more generally, but it is clear that previous research has not fully recognized the importance of both this specific DM and of AAL more generally to the traditional DM system of the South. In addition, we find that DMs are used across the US without any limitation in their form, which we consider to be further evidence of the productivity of these forms in General American English.

Third, we believe our findings provide evidence that is relevant to the debate over the origins of DM in American English. Most notably, we believe our finding that these forms are relatively common on Twitter in areas with relatively high African American populations in the Deep South, and relatively uncommon on Twitter in Appalachia, challenges the generally accepted theory that DMs were imported into American English by Scots-Irish settlers. Rather, we believe our results offer support for an alternative theory, one that has always been dismissed in previous research, namely that DMs are an American innovation, and more specifically an innovation that began with African Americans in the South. Although it seems likely that we will never know for sure how DMs first emerged, given the lack of a historical record, we believe this African American theory of DM origins should be seriously considered alongside the standard Scots-Irish theory in future research. More generally, we believe our findings add to the increasing amount of empirical evidence that shows what a remarkably strong influence AAL has had on American English and, by extension, the English language worldwide.

In addition to these theoretical contributions, we believe our study also provides a strong foundation for future research on DMs in areas that have been outside the scope of this paper, at the most basic level, simply by providing a much more extensive and complete list of the DMs found in American English.

First, we believe our results can inform future work on the syntax of DMs, which has been one of the most active areas in DM research, especially in the generative tradition, where there has been considerable debate on how best to account for these constructions. Certainly, the great diversity of DMs we have observed is further evidence against lexicalist accounts, which treat DMs as a small set of idiomatic expressions [24]. The main debate, however, is whether DMs should treated as a sequence of two modals or if the first form should be treated as adverb, with the adverb-based analysis generally being preferred as it does not involve two tensed verbs in the same clause [5]. Although the productivity in DM formation that we have observed could perhaps be interpreted as evidence that DMs are best analyzed as combinations of two modals verbs, ultimately we believe our results cannot resolve this debate. It is notable, however, that the main attempt to incorporate DMs as sequences of two modals into a generative framework places absolute constraints on the type of modals that can occur in first and second position [6], which is not consistent with our findings. We therefore hope our results will encourage future research on the syntax of DMs, especially to directly account for the productivity in DM formation we have observed. We also believe that DMs can perhaps be better explained through usage-based approaches to grammar, especially Construction Grammar.

Second, we believe our results can inform future research on the meaning of DMs from various perspectives. Given the large number of DM types and tokens we have identified, all of which we make available for future researchers, we hope this study will encourage more detailed analysis of the semantics of DMs. For example, it is unclear to what extent DM choice is affected by the nature of both the subject and the verb of the sentence in which they are embedded. More generally, it is unclear what is the range of meanings that can be expressed by the large numbers of DMs we have observed and how these meanings are related to each other. The pragmatics of DMs, which we have not considered in this paper, is also an area of considerable interest and one where the large inventory of DMs we have provided will hopefully be of value. For example, our results can provide a basis for exploring whether there are specific communicative needs and functions that different DMs fulfill in natural discourse, including across different registers, compared both to each other and more standard constructions, including true adverb–modal sequences. Notably, pursuing these types of questions will likely require the examination of DM use in extended discourse, which was not possible with the dataset we have analyzed in this study; however, given that we have shown that DMs are remarkably common in social media, longer conversation extracted from social media containing DMs could be a basis for such research.

Third, we believe our results can inform future research on the sociolinguistics of DMs, including their social meaning. Most notably, now that we have a better understanding of the inventory of Southern DMs, and now that we have observed variation in DM usage across social groups in the South, more focused research can be conducted to better describe and explain DM use across society and how DM are used by Americans to express their social identity. In addition, as we have argued at length in this paper, we also believe this type of computational sociolinguistic research offers our best opportunity to understand the origins of DMs, perhaps not just in American English, but in English more generally.
